# Development of a framework for increasing asthma awareness in Chitungwiza, Zimbabwe

**DOI:** 10.1186/s40733-019-0052-2

**Published:** 2019-10-29

**Authors:** Pisirai Ndarukwa, Moses J. Chimbari, Elopy N. Sibanda

**Affiliations:** 1 College of Health Sciences, School of Nursing and Public Health, University of KwaZulu Natal, 1st Floor, George Campbell Building, Howard College Campus, UKZN, Durban, 4000 South Africa; 2 Asthma, Allergy and Immune Dsyfunction Clinic, 113 Kwame Nkrumah Ave, Harare, Zimbabwe

**Keywords:** Asthma, Asthma awareness, Framework, Chitungwiza, Zimbabwe

## Abstract

**Background:**

Asthma accounts for significant global morbidity and health-care costs. It is still poorly understood among health professionals and the general population. Consequently, there are significant morbidity and mortality rates throughout the globe. The aim of this study was to develop a framework to increase asthma awareness at Chitungwiza Hospital, Zimbabwe.

**Methods:**

A modified Delphi model was used to collect data to develop a framework for increasing asthma awareness. At baseline (round 1) in-depth interviews with 44 medical doctors were carried out to understand the level of asthma awareness. Round 2 data collection was in the form of a workshop involving a total of 15 doctors, 30 nurses, four public relations officers, and two health education and promotion officers. The same participants who were engaged in round 2 were also involved in the third round where consensus was achieved by the health professionals.

**Results:**

Our study showed that awareness to asthma among health care providers was affected by mimicry of symptoms and lack of continuous education on asthma. Our study showed lack of Information Education and Communication (IEC) material and lack of use of bulk messages affected asthma awareness. Our study showed that clinical meetings on asthma, having asthma training manuals, (IEC) materials and guidelines for asthma diagnosis and management could improve health care providers’ awareness of asthma. Bulk messages on asthma through network providers, social media and bill boards, commemorating world asthma day, edutainment, asthma ambassadors and multimedia were suggested as means of improving awareness of asthma among the public.

**Conclusion:**

We concluded that awareness of asthma can be improved using a framework. Such a framework ultimately improves the quality of asthma care.

## Background

Asthma accounts for significant global morbidity and health-care costs. It is defined as a chronic inflammatory disorder of the small airways whose pathogenesis is linked to variable structural changes that affect both children and adults of all ages [1] Allergic asthma is associated with inhalation of an allergen that stimulate immune response [2].. Despite the high global mortality rate due to asthma, it is still not fully understood among health professionals and the general population [3].

Health messages that reach the entire spectrum of stakeholders at once have been reported to raise awareness of patients, caregivers and health facility managers and policymakers [4]. However, to our knowledge there are no frameworks to ensure that the health messages for asthma reach the health care workforce and patients. Knowledge of asthma symptoms and signs enhances the ability of the health care workers and patients to take appropriate health care decisions [5]. It has been reported that effective asthma control in patients and the population at large depends on their ability to recognise the range of factors influencing the management of the disease [6].

A review of strategies for strengthening asthma education to improve disease control by Clark, et al. [5], reported that family, clinicians, friends, neighbours and work or school mates in the patient’s social environment could act as barriers to effective asthma control due to lack of knowledge about the disease. The health care providers were reported to lack ability to effectively identify asthma cases and educate patients on asthma control [7].

Asthma management techniques including patient education were found not to be sensitive or relevant to culture and language thus causing some difficulties in some ethno cultural communities and minority groups [8]. Therefore, there is need to design a framework which is culturally and language sensitive in order to improve knowledge across different groups of people (patients, communities and health professionals) about asthma [9].

Rosas-Salazar, et al. [10], showed that poor health literacy is an important barrier to asthma knowledge among children and adults. Another study in Zambia showed that only a few health workers were trained in asthma management and they still could not clearly identify asthma cases [11]. Another study in South Africa revealed that health care workers could not distinguish between asthma and chronic obstructive pulmonary disease [12]. This indicates the importance of improving awareness and knowledge of asthma among health care workers for better management of the disease.

Apathy for participation in refresher asthma management courses targeting health care workers has been cited as a contributory factor for poor asthma management [13]. Most health workers are unaware of and do not follow the international guideline on asthma management [13]. This is indicative of the knowledge gap on asthma that exists among health workers. Hence, the need for establishing an asthma awareness framework that may help practitioners and the general public to have more knowledge on asthma management.

The aim of this study was to develop a framework for asthma awareness to improve the ability to recognise asthma within the population and amongst health care providers. The framework is intended to enhance the general public’s knowledge about asthma to make them pro-active in seeking health care for asthma. For health care workers the framework will improve their ability to find cases through appropriate diagnosis informed by the framework.

## Methods

### Study design

**Fig. 1:**
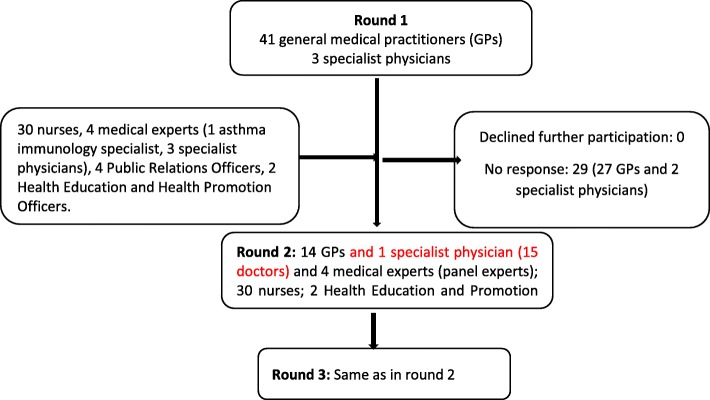
Flowchart of the process of Delphi model showing study participants at each round of participation

We used the modified three stage Delphi method to develop an awareness framework for asthma [14, 15]. The modified process involved three rounds of data collection intended to distil knowledge and build reliable consensus regarding an awareness framework for asthma among health care providers and the public. Figure 1 presents a flowchart of the participants of the three rounds. All the first round participants (doctors) were invited to participate in the final two rounds. In addition, nurses, public relations officers, health education and health promotion officers and the medical experts (specialist doctors) who formed the panel experts were added for rounds 2 and 3 (Fig. 1).

#### Round 1

We aimed to obtain all potentially significant items for the asthma awareness framework. We conducted in-depth interviews with key informants (doctors who were in general practice in medicine and specialist doctors in medicine involved in asthma care). The participants included 41 general doctors and 3 specialist physicians who were based at Chitungwiza Central Hospital. These doctors were briefed on the study aim and objectives. Consent was sought from them to participate in a face to face interviews. The interview solicited for information on the perceived definition and clinical presentation of asthma, difficulties they faced in asthma diagnosis and management; and challenges which they thought were experienced by the health care team and public with regards management of asthma.

#### Round 2

Fifteen doctors and 30 nurses who worked in the medical departments at the hospital, 2 health promotion and education officers (1 working at Chitungwiza Central Hospital (CCH) and the other one working for Chitungwiza City Health Department (CCHD)); and 4 public relations officers (3 working at CCH and 1 working at CCHD) participated in the round 2 data collection phase. This was done through a workshop convened as a follow-up to the first round of data collection. This workshop was led and facilitated by medical experts in asthma care.

Participants at the workshop were divided into two; Group 1 focused on the asthma awareness among the health care providers and Group 2 focused on the asthma awareness among the public.

#### Round 3

In this round of data collection, a panel of medical experts led the process. The two groups were asked to present information on what they believed should be included in the framework for asthma awareness targeting the health care providers and public. Each group presented and a plenary discussion moderated by the experts followed. The objective of the discussion was to modify and adopt information on group assignments (see Fig. 1).

### Data analysis

Data collected in round 1 using in-depth interviews was constructed into consensus statements. The subsequent rounds were done to solidify the presence of consensus. Research findings were reported in thematic format.

## Results

### Round 1 results

In the initial round of the study, results of the in-depth interviews resulted in three thematic areas which were (i) lack of awareness of asthma among some healthcare providers, (ii) inability to diagnose asthma, and (iii) ways to enhance awareness of asthma. These themes are summarized in (Additional file 1: Table S1).

#### Theme 1: asthma awareness among health care providers

The respondents interviewed in this phase indicated there was a general lack of awareness of asthma and it’s symptoms among healthcare providers. Some of the sentiments expressed by the interviewees are indicated in Table 1. Results indicated that respondents felt that health care providers lacked knowledge of asthma presentation, and were inexperienced especially in relation to the ability to tie up presenting complaints and the history that patients provided.

**Table 1:** Summary of themes for the framework of awareness for asthma

Theme	Population Affected/targeted	Possible causes	Proposed solutions	Examples
Lack of asthma awareness	Healthcare providers	• Lack of clinical education • Inexperience	• Refresher courses • Clinical mentoring	*We do not have guidelines to have on asthma diagnosis and this affect our awareness to asthma diagnosis and treatment* (…doctor, Interview 1)
	Patients	• Lack of information, educational and communication (IEC) materials • Lack of health education and health promotion • Misconceptions about asthma	• Strengthening of health promotion • Provision of targeted health messages on asthma	
	General population	• Stigma associated with asthma • Cultural beliefs	• Strengthening health promotion activities	
Inability to diagnose asthma	Healthcare providers	• Lack of training • Lack of appropriate equipment • Comorbidity of asthma with other respiratory conditions • Asthma symptoms mimics other respiratory symptoms.	• Targeted training • Refresher courses • Provision of diagnostic tools • Provision of guidelines for the management of asthma	*I am from school and there is nothing embarrassing as failing to arrive at a conclusive diagnosis…because of lack of resources for diagnosing* (Male doctor, Interview 2). *I end up just giving a provisional diagnosis yet I am sure I will not arrive at an appropriate asthma diagnosis because even if I order tests, these will not be performed…we do not have even basic equipment such as a spirometer to confirm our suspicion* (Male doctor, Interview 3) *What a challenge! To be unable to arrive at a simple diagnosis because simple things like Peak Expiratory Flow Meters are not present* (Senior doctor, Interview 4).
Enhancing awareness of asthma	Health care providers	• Lack of clinical meetings • Lack of IEC material	• Clinical Meetings • In-house training • IEC materials	…*importance of mobile applications with asthma clinical data for health care providers as a way of improving awareness for asthma among the health care providers...*
	Patients and the general public	• Lack of public health awareness programmes on asthma	• Use of bill boards • Bulk messages that are transmitted through network providers • Using asthma champions • Commemoration of World Asthma Days	

##### Asthma awareness among patients and the general public

Our study revealed that interview respondents concurred on the need for creating awareness for asthma among the public through the use of bill boards, bulk messages that are transmitted through network providers, use of asthma champions, social media, commemorations of world asthma days and use of multimedia (Table 1). Healthcare providers highlighted the need to inform the public of the risk factors for asthma such as history of allergies and atopy, and family history of asthma. The doctors indicated the need for symptoms of asthma to be packaged IEC materials which would be widely distributed into the communities as a way of increasing awareness of asthma.

#### Theme 2: inability to diagnose asthma

Results from the in-depth interviews revealed healthcare providers were having difficulties diagnosing asthma (Table 1) and this was mainly due to lack of diagnostic equipment such as Peak expiratory flow meters, spirometers, reagents for RAST tests, Bronchial provocation tests and non-availability of definitive lung function for asthma diagnosis. Additionally, clinicians reported that the presence of comorbidities such as emphysema and COPD posed challenges to some healthcare providers when diagnosing asthma. Some respondents reported having difficulties in differentiating asthma from heart diseases which affected their clinical judgement relating to asthma diagnosis.

#### Theme 3: ways to enhance awareness

Respondents suggested a number of ways to create and improve awareness of asthma (see Table 1). Interviewees emphasized on the need for an awareness framework for health care providers and for the public. Also raised during the interviews was the need for current asthma algorithms for diagnosis in the consultation rooms for health care providers as a way of creating and enhancing asthma awareness among clinicians. There was consensus among all doctors who said clinical meetings would improve awareness of asthma. They also highlighted the need for constant workshops on recent developments on asthma diagnosis and management. Communication with other health care workers involved in asthma diagnosis and management such as nurses was also important.

### Round 2 results

The themes emerged from round one of the study were presented to respondents during round 2. Results indicated that the viewpoints from round 2 were not at variance with the initial in-depth interviews which had been conducted with the doctors. The panel of experts who moderated during the presentation, asked participants how they expected the awareness framework to look like. All participants agreed that the asthma awareness framework should benefit both the health care professionals and the public.

They suggested that asthma awareness framework should be implemented through; Use of Clinical workshops (health professional), Training manuals, and Information Education and Communication (IEC) materials for consultation rooms (Posters). Bulk messages through mobile telecommunications network providers such as NetOne, Telecel and Econet. Use of champions (Asthma ambassadors such as popular musicians), Social media, Bill boards, Commemorations of world asthma day (take note of seasonality issues), (Ministries of health, environment), edutainment (road shows, dramas), Multimedia (Bill boards, Televisions and radio adverts on asthma, Pamphlets).

### Round 3

**Fig. 2:**
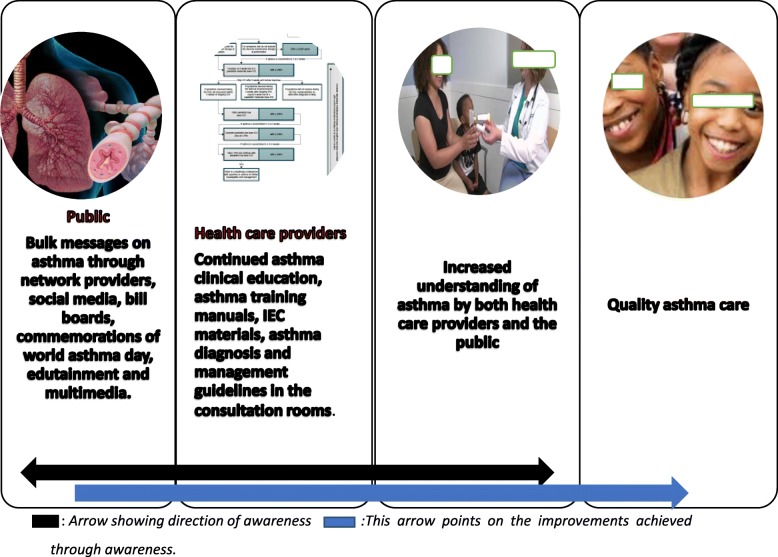
Asthma awareness framework© (P Ndarukwa^1^, M J Chimbari^1^ and E N Sibanda^2^, 2019)

A draft framework was developed by the authors which was then presented to the panel of experts for adoption. The participants adopted the asthma awareness framework (Fig. 2).

## Discussion

Using the modified Delphi model (in-depth interviews, group works and group presentations, and panel discussion) we developed an asthma awareness framework. The study consisted of three phases leading to consensus on the asthma awareness framework. All the participants for all phases suggested that clinical meetings on asthma, having asthma training manuals, IEC materials and guidelines for asthma diagnosis and management could improve awareness of asthma by health care providers. It was also suggested that bulk messages on asthma through network providers, social media and bill boards, commemorating world asthma day, edutainment, asthma ambassadors and multimedia may improve awareness of asthma among the public. We concluded that efforts to improve awareness for asthma should involve all the stakeholders.

### Awareness of asthma among health care providers

Holding of the clinical meetings on asthma and having training manuals were suggested as means for improving awareness of asthma among health care professional. These strategies address the lack of asthma knowledge by care providers who may experience difficulties in diagnosing and managing asthma. Our study findings are in agreement with other studies [16–18] that have underscored the importance of education sessions to improve the awareness of asthma. Kang, et al. [18], reported that effective educational tools are important for increasing adherence to asthma guidelines and clinical improvement of asthma patients.

Information, Education and Communication materials and guidelines for asthma diagnosis and management were also reported in our study as some of the strategies to improve awareness of health care professions in asthma. Other studies have also shown that IEC improves people’s health by increasing their awareness and knowledge; and changing attitudes and behaviours [19–21]. Furthermore, guidelines for asthma diagnosis and management are reported as effective means by which health care professionals can learn rapidly and amass vast knowledge and skills in asthma, thus contributing to their awareness of the condition [21].

### Awareness of asthma among the public

This study has indicated that the public’s awareness of asthma can be increased by use of bulk messages through network providers. Sharing asthma messages is a key component towards achieving asthma awareness and this promote the community to be proactive in dealing with cases of asthma [22]. The use of network providers can rapidly spread messages about asthma ensuring that a wide audience is reached at one goal thus improving awareness. Bill boards were also reported in our study to be effective means for public awareness of asthma. This has been revealed in other studies that reported bill boards as effective tools for mass awareness [23, 24]. Other studies have suggested that messages on the bill board must be displayed with pictures that can easily be comprehended by the general public [23, 25].

In this study we have learnt that multimedia can improve asthma awareness to the public. This was also reported in other studies that demonstrated that local news media can be an effective partner for disseminating asthma messages [26, 27]. Use of local television and radio-talk shows hosting asthma care professionals to address community asthma awareness, thus improving understanding the public about asthma [26]. Our study further asserted that edutainment in the form of drama and role plays can improve asthma awareness among the public. This is consistent with findings from other studies that have reported extensively on the role of edutainment [28–30].

Social media was suggested, in our study, as important for improving asthma awareness. This is also reflected in another study indicating that social media is increasingly being used as a tool to disseminate information on diseases to increases public awareness [31].

Consistent with the World asthma report [31, 32], our study suggests that commemoration of world asthma day can improve asthma awareness among the public.

## Conclusion

We developed a framework for asthma awareness appropriate for health care providers and the public. We recommend the adoption of the framework for asthma awareness of the Ministry of Health and Child Care, Zimbabwe.

## Supplementary information

**Additional file 1: Table S1**. Summary of themes for the framework of awareness for asthma.

## Data Availability

The datasets used and/or analysed during the current study are available from the corresponding author on reasonable request.

## Abbreviations

CCH: Chitungwiza Central Hospital
CCHD: Chitungwiza City Health Department
COPD: Chronic Obstructive Pulmonary Disease
GPs: General Practitioners
IEC: Information, Education and Communication
PEF: Peak Expiratory Flow
RAST: Radioallergosorbent testing
UKZN: University of KwaZulu-Natal
