# Supplementary material for: Development of a framework for increasing asthma awareness in Chitungwiza, Zimbabwe
Source: Asthma Res Pract. 2019 Oct 29;5:4. doi: 10.1186/s40733-019-0052-2 (PMC6820964; doi:10.1186/s40733-019-0052-2)
Supplement: Supplementary file 1 — Additional file 1: Table S1. Summary of themes for the framework of awareness for asthma. [file 40733_2019_52_MOESM1_ESM.docx]

**Table S1: Summary of themes for the framework of awareness for asthma.**

| **Theme** | **Population Affected/targeted** | **Possible causes** | **Proposed solutions** | **Examples** |
| --- | --- | --- | --- | --- |
| **Lack of asthma awareness** | Healthcare providers | - Lack of clinical education - Inexperience | - Refresher courses - Clinical mentoring | *We do not have guidelines to have on asthma diagnosis and this affect our awareness to asthma diagnosis and treatment* (…doctor, Interview 1) |
|  | Patients | - Lack of information, educational and communication (IEC) materials - Lack of health education and health promotion - Misconceptions about asthma | - Strengthening of health promotion - Provision of targeted health messages on asthma |  |
|  | General population | - Stigma associated with asthma - Cultural beliefs | - Strengthening health promotion activities |  |
| **Inability to diagnose asthma** | Healthcare providers | - Lack of training - Lack of appropriate equipment - Comorbidity of asthma with other respiratory conditions - Asthma symptoms mimics other respiratory symptoms. | - Targeted training - Refresher courses - Provision of diagnostic tools - Provision of guidelines for the management of asthma | *I am from school and there is nothing embarrassing as failing to arrive at a conclusive diagnosis…because of lack of resources for diagnosing* (Male doctor, Interview 2).  *I end up just giving a provisional diagnosis yet I am sure I will not arrive at an appropriate asthma diagnosis because even if I order tests, these will not be performed…we do not have even basic equipment such as a spirometer to confirm our suspicion* (Male doctor, Interview 3)  *What a challenge! To be unable to arrive at a simple diagnosis because simple things like Peak Expiratory Flow Meters are not present* (Senior Clinician, Interview 4). |
| **Enhancing awareness of asthma** | Health care providers | - Lack of clinical meetings - Lack of IEC material | - Clinical Meetings - In-house training - IEC materials | …*importance of mobile applications with asthma clinical data for health care providers as a way of improving awareness for asthma among the health care providers...* |
|  | Patients and the general public | - Lack of public health awareness programmes on asthma | - Use of bill boards - Bulk messages that are transmitted through network providers - Using asthma champions - Commemoration of World Asthma Days |  |
